# Notch Signaling-Induced Oscillatory Gene Expression May Drive Neurogenesis in the Developing Retina

**DOI:** 10.3389/fnmol.2019.00226

**Published:** 2019-09-19

**Authors:** Dmitry Ivanov

**Affiliations:** ^1^Department of Ophthalmology, Bascom Palmer Eye Institute, University of Miami Miller School of Medicine, Miami, FL, United States; ^2^Department of Microbiology and Immunology, University of Miami Miller School of Medicine, Miami, FL, United States

**Keywords:** retinal neurogenesis, retinal progenitor cells, notch signaling, lateral inhibition, oscillatory gene expression, retinal phenotypes

## Abstract

After integrating classic and cutting-edge research, we proposed a unified model that attempts to explain the key steps of mammalian retinal neurogenesis. We proposed that the Notch signaling-induced lateral inhibition mechanism promotes oscillatory expression of Hes1. Oscillating Hes1 inhibitory activity as a result leads to oscillatory expression of Notch signaling inhibitors, activators/inhibitors of retinal neuronal phenotypes, and cell cycle-promoting genes all within a retinal progenitor cell (RPC). We provided a mechanism explaining not only how oscillatory expression prevents the progenitor-to-precursor transition, but also how this transition happens. Our proposal of the mechanism posits that the levels of the above factors not only oscillate but also rise (with the exception of Hes1) as the factors accumulate within a progenitor. Depending on which factors accumulate fastest and reach the required supra-threshold levels (cell cycle activators or Notch signaling inhibitors), the progenitor either proliferates or begins to differentiate without any further proliferation when Notch signaling ceases. Thus, oscillatory gene expression may regulate an RPC’s decision to proliferate or differentiate. Meanwhile, a post-mitotic precursor’s selection of one retinal neuronal phenotype over many others depends on the expression level of key transcription factors (activators) required for each of these retinal neuronal phenotypes. Because the events described above are stochastic due to oscillatory gene expression and gene product inheritance from a mother RPC after its division, an RPC or precursor’s decision requires the assignment of probabilities to specific outcomes in the selection process. While low and sustained (non-oscillatory) Notch signaling activity is required to promote the transition of retinal progenitors into various retinal neuronal phenotypes, we propose that the lateral inhibition mechanism, combined with high expression of the BMP signaling-induced Inhibitor of Differentiation (ID) protein family, promotes high and sustained (non-oscillatory) Hes1 and Hes5 expression. These events facilitate the transition of an RPC into the Müller glia (MG) phenotype at the late stage of retinal development.

**GRAPHICAL ABSTRACT:**
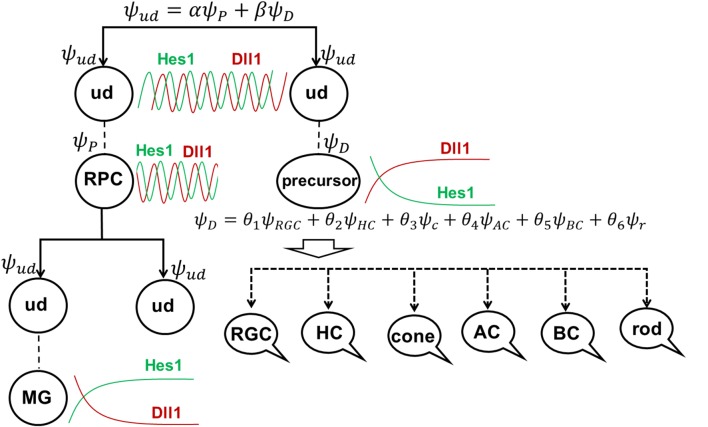


## Introduction

The number of people suffering from retinal diseases is projected to increase significantly in the coming decades, especially as the population of elderly patients continues to grow (Rosenberg and Sperazza, 2008; Akpek and Smith, 2013). These diseases lead to progressive retinal damage and, ultimately, to blindness; yet they remain difficult or impossible to treat, even with the surfeit of sophisticated technologies and therapies available to the modern ophthalmologist. Regenerative medicine provides unique opportunities to restore, or even replace, tissue that has been damaged or completely lost (Nirenberg and Pandarinath, 2012; Dhamodaran et al., 2014; Schwartz et al., 2015; Vandenberghe, 2015; Jorstad et al., 2017; Holmes, 2018; Llonch et al., 2018; Yao et al., 2018). However, to apply regenerative approaches to retinal tissue, we need to understand the intricate molecular mechanisms that underlie retinal neurogenesis. The relevant literature contains hundreds of articles that precisely identify the molecular mechanisms that regulate different aspects of retinal neurogenesis. Nevertheless, the current scientific understanding can be likened to a jigsaw puzzle strewn across the floor—individual “pieces” are intact, but remain unassembled into a coherent whole. We argue here that the concept of Notch signaling-induced oscillatory gene expression in retinal progenitors allows assembly of these “pieces” into the complete model of neurogenesis we propose for the developing retina.

**Figure 1:**
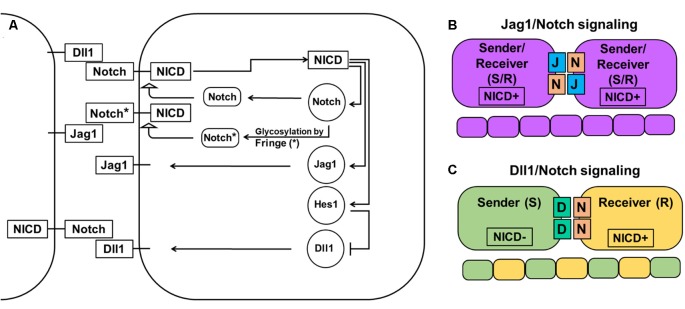
Notch-Delta-Jagged signaling. **(A)** The Notch pathway is activated when the Notch receptor of one cell interacts with the ligand of a neighboring cell leading to cleavage of the Notch intracellular domain (NICD) and its release into the cytoplasm. NICD acts as a transcription factor and controls the expression of many genes, including Notch receptors and ligands. However, while it directly activates the expression of Notch receptors, the Jag1 ligand, and transcription factor Hes1 (which acts as an inhibitor of any gene expression), it indirectly inhibits Dll1 expression through Hes1. The special family of fringe proteins (Lfng and Mfng) increases the Notch receptor’s affinity to bind to Dll1 and decreases it for Jag1, thus promoting lateral inhibition over lateral induction. **(B)** Jag1/Notch signaling forms a double positive feedback loop between the two neighboring cells and forces them to adopt the same fate/phenotype: high expressions of the Notch receptor, Jag1, Hes1, and low Dll1 expression. The Jag1/Notch cells both send (due to Jag1 ligands) and receive (due to Notch receptors) signals [a so-called hybrid Sender/Receiver (S/R) phenotype]. The mechanism that promotes this phenotype is known as lateral induction. **(C)** Dll1/Notch signaling causes a double-negative feedback loop between the two neighboring cells promoting them to acquire two opposite fates/phenotypes (a “salt-and pepper” pattern): Sender (S; high Dll1; low Notch, Hes1, and Jag1) and Receiver (R; high Notch and Hes1; low Dll1 and Jag1). The mechanism that promotes the two opposite fates/phenotypes is known as lateral inhibition.

The Notch signaling pathway consists of four transmembrane receptors (Notch1–4) and five ligands (Dll1, Dll3, Dll4, Jag1, and Jag2). Notch1 and Notch2 receptors and Dll1 and Jag1 ligands play important roles in a majority of tissues during development and in adulthood, while Notch4 and Dll4 are mostly important during angiogenesis (Hirata et al., 2002; Baek et al., 2006; Ohsawa and Kageyama, 2008; Benedito et al., 2009; Nelson et al., 2009; Andersson et al., 2011; Shimojo et al., 2011; Wang et al., 2011; Kume, 2012; Taylor et al., 2014; Boareto et al., 2015a,b; Dvoriantchikova et al., 2015; Kakuda and Haltiwanger, 2017). Notch signaling plays a critical role in determining cell fate and tissue patterning (Figure 1). Notch signaling, through Dll1 and Jag1, leads to different outcomes. Dll1/Notch signaling, due to a lateral inhibition mechanism, promotes a “salt-and-pepper” pattern when neighboring cells adopt two different fates [sender (S) and receiver (R); Figure 1; Boareto et al., 2015a,b; Kakuda and Haltiwanger, 2017]. Jag1/Notch signaling, due to a lateral induction mechanism, drives neighboring cells to adopt a similar fate—hybrid S/R (Boareto et al., 2015a,b; Kakuda and Haltiwanger, 2017). Thus, while lateral induction promotes only one phenotype, Notch/Dll1-mediated lateral inhibition allows generation of the huge variety of cell types that we observe in the retina. In the developing mammalian retina, Notch signaling is necessary for the maintenance of a proliferating retinal progenitor cell (RPC) population and the prevention of untimely progenitor differentiation (Bao and Cepko, 1997; Furukawa et al., 2000; Jadhav et al., 2006a,b; Ohsawa and Kageyama, 2008; Mizeracka et al., 2013; Cepko, 2014). Inhibition of Notch signaling causes premature differentiation of RPCs into different types of retinal neurons, while Notch signaling upregulation promotes RPC differentiation into Müller glia (MG; Furukawa et al., 2000; Jadhav et al., 2006a,b; Ohsawa and Kageyama, 2008; Mizeracka et al., 2013). To initiate lateral inhibition, Dll1 (a highly expressed Notch ligand in the developing retina) located on a differentiating cell’s surface *trans*-activates a Notch receptor in an adjacent progenitor. *Trans*-activation causes cleavage of the Notch receptor protein’s intracellular domain (NICD), which then travels to the nucleus to form a complex with the DNA-binding protein RbpJ. The NICD/Rbpj complex activates the expression of inhibitors of neuronal differentiation, such as Hes1 and Hes5, thereby keeping the *trans*-activated cell in an undifferentiated progenitor state or promoting MG differentiation during the late stage of retinal development (Ohsawa and Kageyama, 2008). If Notch signaling is not activated, the consequent absence of Hes1 and Hes5 activity allows the expression of proneuronal transcription factors that induce the expression of Notch ligands and proneuronal genes, followed by differentiation into a variety of retinal neuronal phenotypes (Ohsawa and Kageyama, 2008). However, vast amounts of information suggest that such a chain of events is most likely caused by, not sustained (high or low) gene expression, but oscillatory gene expression—which is easily provoked by the Notch-mediated lateral inhibition mechanism (Kageyama et al., 2008b; Shimojo et al., 2008).

## The Notch-Dependent Oscillatory Gene Regulatory Network (GRN) Prevents the Differentiation of Progenitors Into Retinal Phenotypes, but Creates the Foundation for RPC Differentiation Into all Retinal Neuronal Phenotypes

**Figure 2:**
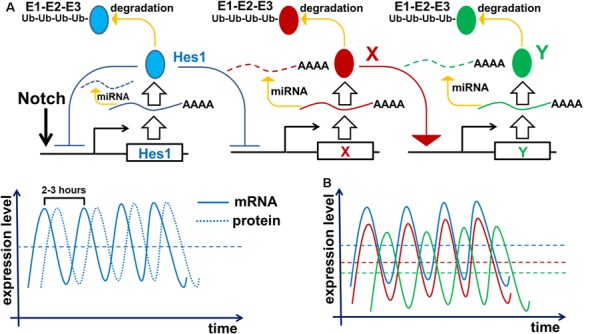
Negative feedback loops are a common feature of gene networks. Negative feedback can drive oscillatory gene expression depending on the gene’s unique expression dynamics (time delay from transcription to translation, protein and mRNA half-lives, and efficiency of miRNA and proteasome degradation pathways). **(A)** Notch signaling activates expression of the Hes1 gene, causing gradual accumulation of Hes1 mRNA. Since there is a delay between Hes1 RNA transcription and translation, Hes1 mRNA levels will initially be much higher than Hes1 protein levels. But because Hes1 protein negatively regulates its own production by binding to, and blocking, the Hes1 promoter, growing Hes1 protein levels cause Hes1 mRNA production to fall. The instability of Hes1 mRNA (degraded *via* a miRNA pathway) contributes to further Hes1 mRNA decline. Increasing Hes1 protein levels continue to dampen Hes1 transcription until it ceases altogether. But since Hes1 proteins are also unstable (degraded *via* the 26S proteasome pathway), Hes1 protein levels eventually reach a maximum and then begin to decline, allowing renewed Hes1 transcription. The delay between Hes1 transcription and mature Hes1 protein production enables Hes1 protein levels to fall while Hes1 mRNA levels rise again. Repetition of this cycle drives Hes1’s oscillatory expression dynamics. It should be noted that Hes1 mRNA and protein levels oscillate with a phase shift in regards to each other. **(B)** The aforementioned mechanism of Hes1’s oscillatory expression may be applied to all genes whose expression is inhibited by Hes1 (Neurog2, Ascl1, Atoh7, Otx2, etc.; depicted here as gene X). Thus, expression of such genes should also oscillate. In this regard, if the expression of gene X oscillates, then the expression of genes activated by gene X (gene Y; e.g., Neurog2 and Ascl1 activate Dll1 expression) may also oscillate. The oscillatory expression of genes X and Y should be phase-shifted with respect to each other since products of gene X (mRNA and protein) must first be present to initiate the expression of Y.

To inhibit gene transcription, the Notch signaling-activated transcription factor Hes1 binds to the N-box and C-site (Takebayashi et al., 1994; Van Doren et al., 1994; Chen et al., 1997; Hirata et al., 2002; Bai et al., 2007; Kageyama et al., 2008a). The Hes1 promoter contains three N-box regulatory elements that Hes1 uses to inhibit its own expression (Takebayashi et al., 1994; Hirata et al., 2002; Bai et al., 2007; Kageyama et al., 2008a). Such negative feedback loops are an extremely common feature of many gene regulatory networks (GRNs) throughout the tree of life (Kageyama et al., 2007b; Pigolotti et al., 2007; Lahav, 2008). In eukaryotic cells, inhibition of gene expression by negative feedback loops implies the existence of delays in time to inhibition (Figure 2). The length of such delays is determined by the time required for transcription, intron splicing, translation, and post-translational modifications; all of which are unique for each gene. Theoretical analysis and experimental data indicates that delayed negative feedback loops should drive oscillatory gene expression if the delay is longer than the half-lives of the gene’s mRNA and protein (Figure 2; Hirata et al., 2002; Jensen et al., 2003; Monk, 2003; Shimojo et al., 2008; Swinburne et al., 2008; Takashima et al., 2011; Tiana and Jensen, 2013). Given that such conditions are very common, oscillatory gene expression should occur quite frequently. Mathematical models of Hes1 expression using experimental data of the time delays and half-lives of Hes1 mRNA and protein have demonstrated that Hes1 oscillates with a period in the range of 2–3 h (Jensen et al., 2003; Monk, 2003; Zeiser et al., 2007). These theoretical data are in good agreement with experimental observations (Hirata et al., 2002). High and sustained Hes1 expression prevents the expression of many genes required for neuronal phenotypes as well as genes that regulate and promote cell proliferation (Castella et al., 2000; Baek et al., 2006; Kageyama et al., 2008a; Ohsawa and Kageyama, 2008; Shimojo et al., 2008; Noda et al., 2011). However, oscillatory Hes1 gene expression provokes oscillatory expression of genes that are normally inhibited by Hes1: as Hes1 expression oscillates, mRNA expression of target genes follows the inverse pattern; their mRNA expression is permitted at moments when the Hes1 protein level is low, and prevented when the Hes1 protein level reaches its maximum (Figure 2). Since genes directly inhibited by Hes1 also activate or inhibit the expression patterns of their own groups of target genes, oscillatory Hes1 expression acts as an engine, propelling oscillatory expression across numerous interacting genes; we deem this an oscillatory GRN. Thus, oscillatory Hes1 activity may even allow the expression of many antagonistic genes including Notch signaling inhibitors, activators/inhibitors of retinal neuronal phenotypes, and cell cycle-promoting genes in the same progenitor. Within the field of development, oscillatory expression has been principally championed by Dr. Kageyama, a celebrated scientist from the University of Kyoto’s Institute for Virus Research. Kageyama et al. (2008b) have repeatedly and convincingly demonstrated that oscillatory expression of Notch-dependent genes and other cell-fate determination factors is required for the maintenance of a progenitor pool (Shimojo et al., 2011; Isomura and Kageyama, 2014; Imayoshi et al., 2015). In his words, “*multipotency is a state of multiple oscillating fate determination factors, while cell fate choice (differentiation) is a process of sustained expression of a single factor*” (Imayoshi et al., 2013). Thus, oscillatory gene expression serves to maintain a population of proliferating progenitor cells, buying time during development to generate enough cells to form a tissue of normal size. Employing the concept of oscillatory gene expression in progenitor cells, Dr. Kageyama suggested that at the earliest stages of tissue development—before any progenitors have begun to differentiate—the observed lateral inhibition mechanism-induced “salt-and-pepper” expression pattern of Notch-dependent genes and other cell-fate determination factors is merely a “snapshot” of oscillatory gene expression in adjusted progenitors (Kageyama et al., 2008b). It is important to note that oscillatory expression of not only inhibitors but also activators of neuronal fate plays an important role in progenitors. Experimental data indicate that the expression of Ascl1 (an activator of neuronal differentiation) oscillates in progenitors, facilitating their proliferation (Imayoshi et al., 2013). By contrast, sustained expression of Ascl1 prevents proliferation and mediates differentiation (Imayoshi et al., 2013).

Oscillatory gene expression is a broadly applicable phenomenon that enables us to explain myriad RPC operations and behaviors. We propose that oscillatory gene expression prevents an RPC’s transition from the proliferative progenitor state to the post-mitotic precursor state, but allows the expression of Notch signaling inhibitors, activators/inhibitors of retinal neuronal phenotypes, and cell cycle-promoting genes—since inhibitory Hes1 (and Hes5, as part of the GRN) activity oscillates from high to low. This hypothesis can now explain the extensive heterogeneity of gene expression observed in individual RPCs (Trimarchi et al., 2008; Cepko, 2014). We can expect randomly collected progenitors to be in different/random phases of oscillatory gene expression, therefore having different/random mRNA and protein levels reflecting the instant (“snapshot”) at which the cells were collected, lysed, and used for analysis. Thus, the observed heterogeneity of gene expression in individual (single) RPCs most likely reflects different phases of oscillatory gene expression in equivalent RPCs. In this regard, as a response to Dr. Cepko’s question about whether the extensive heterogeneity of progenitor gene expression is due to “*intrinsic differences among RPCs or to extrinsic and/or stochastic effects on equivalent RPCs or their progeny*,” we argue that the heterogeneity/variability of RPC gene expression may result from phase-shifted oscillatory gene expression in a small number of equivalent RPC types (Cepko, 2014). Importantly, since oscillatory Hes1 expression may allow the expression of key genes required for all retinal neuronal phenotypes in the same RPC, this progenitor is ready to differentiate into any retinal neuronal phenotype when Notch signaling activity ceases. The decision to differentiate into a particular neuronal phenotype will depend upon the level of factors required for this phenotype (refer to the “*‘Quantum Mechanics’* of Retinal Phenotypes” section).

There is an abundance of direct evidence of oscillatory gene expression in progenitors of the developing brain, during somitogenesis, and in some cell lines (Hirata et al., 2002; Izumo et al., 2003; Nelson et al., 2004; Shimojo et al., 2008; Takashima et al., 2011; Kageyama et al., 2012; Imayoshi et al., 2013; Goodfellow et al., 2014). But to date, no direct evidence of oscillatory expression in the retina has been produced. However, a solid body of indirect data support a model of oscillatory gene expression in RPCs. Our published data and other studies indicate expression of both repressors and activators of neuronal fate specification in RPCs (high and sustained Hes1/Hes5 expression should prevent the expression of such neuronal fate-specific factors). Our findings indicated that, while Hes1 and Hes5 (inhibitors of differentiation) were highly expressed in early RPCs, expression of the pro-neuronal factors Atoh7 and Otx2 was significantly (some 30-fold) higher (Dvoriantchikova et al., 2015). High and steady expression levels of Hes1 have been shown to inhibit the expression of Atoh7 and Otx2 (Brown et al., 1998; Mu et al., 2008; Muranishi et al., 2011; Maurer et al., 2014). Thus, high expression of Atoh7 and Otx2 in early RPCs implies oscillation of Hes1 expression: i.e., Atoh7 and Otx2 should accumulate during the periodic troughs in Hes1 expression and inhibitory activity. In addition, the published observations of Muranishi et al. (2011) suggest that Otx2 and Hes1 protein levels should oscillate with a phase shift in RPCs. While these authors detected some RPCs with exclusively high Otx2 expression and other RPCs with exclusively high Hes1 expression, they also found RPCs that co-expressed these genes at lower levels. Our results are also consistent with two studies from Dr. Reh’s laboratory indicating simultaneous expression of repressors (Notch1, Hes5, Hes1, Hey2, etc.) and activators (Dll1, Dll3, Ascl1, Neurog2, etc.) of neuronal fate specification in RPCs (Nelson et al., 2009, 2011).

Despite the encouraging uniformity of the results above, future studies of oscillatory gene expression in the developing retina must still be carried out and must be done very carefully in order to avoid incorrect conclusions. The simple possibility of phase shifts in expression makes it very difficult to guarantee observation of simultaneous expression of two antagonistic factors, even if a progenitor is indeed expressing both. An optimal approach to studying oscillatory gene expression in the retina will likely involve real-time/time-lapse imaging methods combined with destabilized luciferase reporters (Imayoshi et al., 2013, 2015; Shimojo et al., 2014). However, while this technology can be efficiently used *in vitro*, using it for *in vivo* studies remains exceedingly challenging. We can also propose the use of 10× Genomics technology to perform high-throughput gene expression analysis in tens of thousands of RPCs simultaneously. Since embryonic day (E) 11–14 retinas contain mostly RPCs, cells collected from these retinas, in combination with bioinformatic analysis, can be used to reconstruct the oscillatory Hes1 GRN.

## At Least Two Transcription Factors Are Necessary to Promote Retinal Neuronal Phenotypes

**Figure 3:**
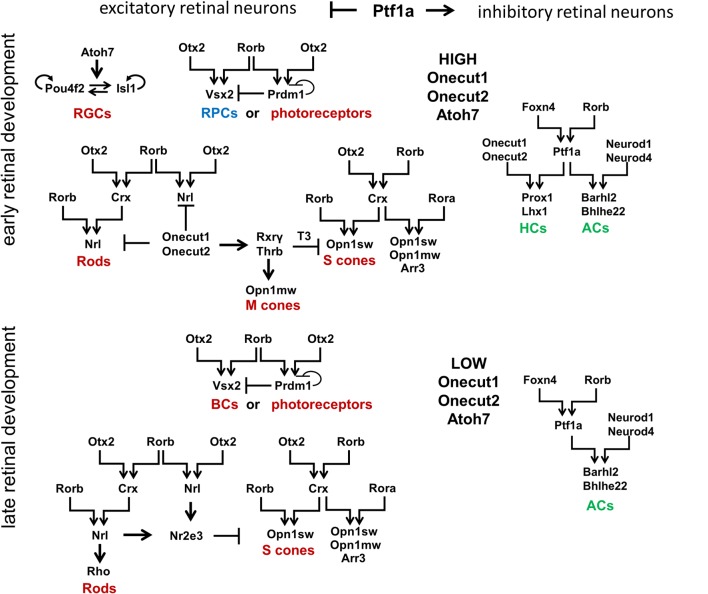
Signaling cascades that regulate development of retinal neuronal phenotypes. Retinal neuronal phenotypes can be classified based on: (1) the time point in which they are generated during retinal development (early-born or late-born); and (2) the release of neurotransmitters that excite (excitatory retinal neurons) or inhibit (inhibitory retinal neurons) the firing of an action potential. While high levels of Onecut1, Onecut2, and Atoh7 are required to generate early-born retinal neurons from RPCs, the transcription factor Ptf1a has been unequivocally shown to function as a molecular “switch” between excitatory and inhibitory fates. It should be noted that Otx2 and Rorb play a special role in the production of all excitatory retinal phenotypes (with the exception of RGCs). Foxn4 and Rorb transcription factors are necessary for the generation of all inhibitory retinal neurons. RGCs, retinal ganglion cells; HCs, horizontal cells; ACs, amacrine cells.

To propose a mechanism that regulates retinal development, we first need to describe known transcription factors that regulate retinal neuronal phenotypes. Certain aspects of retinal development have already been concretely established and will be briefly reviewed here. Retinal development begins with a population of equivalent, proliferating early RPCs having a specific “competence” in generating only early-born retinal neurons [two excitatory types: retinal ganglion cells (RGCs) and cone photoreceptors; and two inhibitory types: horizontal cells (HCs) and a subpopulation of early-born amacrine cells (ACs)], as well as a population of late RPCs (Figure 3; Elliott et al., 2008; Georgi and Reh, 2010; Kohwi and Doe, 2013; La Torre et al., 2013). Meanwhile, late RPCs generate late-born retinal neurons [two excitatory types: rod photoreceptors and bipolar cells (BCs); and one inhibitory type: a subpopulation of late-born amacrine cells (ACs)], as well as the non-neuronal MG. High Atoh7 expression in early RPCs—not late RPCs—promotes differentiation of these progenitors into RGCs at the early stage of retinal development (Figure 3; Brown et al., 1998; Ohsawa and Kageyama, 2008). The transcription factors Onecut1 and Onecut2 redundantly regulate the formation of all early-born retinal neurons, while preventing the generation of the late-born varieties (Figure 3; Emerson et al., 2013; Wu et al., 2013; Sapkota et al., 2014). Meanwhile, excitatory or inhibitory neuronal types depend on the transcription factor Ptf1a, which functions as a molecular “switch” between the two types in the developing retina (Figure 3; Fujitani et al., 2006; Dullin et al., 2007; Nakhai et al., 2007; Jusuf et al., 2011). Ptf1a, in conjunction with Rbpj (a DNA binding protein that can also interact with the intracellular domains of Notch family proteins), inhibits the expression of both Atoh7 and Otx2, thereby facilitating differentiation into ACs (post-mitotic expression of Ptf1a and Neurod1/Neurod4) or HCs (post-mitotic expression of Ptf1a and Onecut1/Onecut2; Figure 3; Fujitani et al., 2006; Dullin et al., 2007; Nakhai et al., 2007; Hori et al., 2008; Jusuf et al., 2011; Lelièvre et al., 2011; Wu et al., 2013). However, the inhibitory fate decision can only be executed if a progenitor contains low levels of activators of excitatory fate (transcription factors such as Atoh7, Vsx2, or Otx2; Jusuf et al., 2011). That is, if excitatory fate activators are not sufficiently repressed, no amount of Ptf1a expression will override the excitatory fate program (Jusuf et al., 2011). Moreover, post-mitotic cells with conditional Ptf1a ablation can be forced to abandon an inhibitory fate for an excitatory one (Fujitani et al., 2006;Jusuf et al., 2011).

The analysis of transcription factors regulating the development of different retinal neuronal phenotypes allows us to posit the retinal GRN (rGRN), which controls the development of all retinal neuronal phenotypes, except RGCs. On the top of this rGRN, there are three highly expressed transcription factors in RPCs: Otx2, Foxn4, and Rorb. Otx2 and Rorb are on the top of the branch regulating the development of excitatory retinal phenotypes, while Foxn4 and Rorb are on the top of the branch regulating the development of inhibitory retinal phenotypes due to activation of Ptf1a expression (Li et al., 2004; Montana et al., 2011; Liu et al., 2013; Fu et al., 2014; Wang et al., 2014). Rorb, thus, combines both of these branches into one united rGRN. Otx2 and Rorb promote Vsx2 (Chx10) expression, preventing RPC differentiation at the early stage of retinal development and promoting the BC phenotype at the late stage (Horsford et al., 2005; Wang et al., 2014). Otx2/Rorb-regulated Prdm1 (Blimp1) is required for rod photoreceptor differentiation at the late stage of retinal development (Wang et al., 2014). However, Prdm1 is highly expressed in early RPCs (Dvoriantchikova et al., 2015). Thus, we propose that Prdm1 is required to promote the different photoreceptor phenotypes (cone or rod) depending on the stage of retinal development (Figure 3). Otx2 and Rorb mediate Crx expression, which promotes the cone phenotype along with Rora, and requires Rorb to mediate Nrl expression in precursors that are followed by the rod phenotype (Swaroop et al., 2010). As we noted above, Ptf1a and Onecut1/Onecur2 promote genes required for HC development, while Ptf1a and Neurod1/Neurod4 are necessary for the AC phenotype. Thus, the rGRN, much like a Galton board (or “bean machine”), contains many levels that direct a precursor to select one of many retinal neuronal phenotypes. However, each level requires at least two transcription factors to promote transition to the next (lower) level. Even the RGC GRN, which stands apart from the general rGRN, still requires the two transcription factors Pou4f2 and Isl1 to promote the RGC fate (Mu et al., 2008; Wu et al., 2015). Since our model of retinal neurogenesis is based on oscillatory gene expression—and understanding that Rora and Rorb are both involved in the body’s circadian clock function—it is important to note that Rora (and possibly Rorb) expression exhibits a circadian oscillation in the retina (Kamphuis et al., 2005;Tosini et al., 2007).

## The Progenitor-to-Precursor Transition Requires a Transition From Oscillatory to Low and Sustained (Non-oscillatory) Notch Signaling Expression; Regulation of Retinal Size and Carcinogenesis by Transition Time

**Figure 4:**
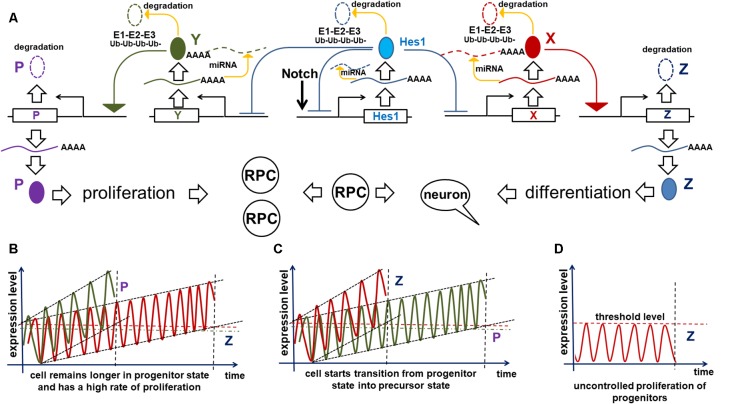
Implication of the oscillatory Hes1 gene regulatory network (GRN) in a progenitor’s decision to proliferate or start differentiation. **(A)** A putative signaling cascade that regulates a progenitor’s decision to proliferate or start differentiation. In our model, the expression of a gene designated as Z is required for Notch signaling inactivation followed by progenitor differentiation. A gene designated as P promotes cell proliferation. Supra-threshold levels of these genes in a short period of time are necessary to activate corresponding processes. Meanwhile, we propose that sub-threshold levels of products of these genes are unstable and quickly degrade without inducing any effect. Suppose that one or more oscillatory Hes1 GRN genes, designated as X (Y), promote Z (P) expression in a progenitor. Suppose that products of X and Y accumulate over time. The moment they reach high enough levels (separate supra-threshold levels for X and Y) to promote sufficiently high production of Z (P), the progenitor ceases Notch signaling, transiting into the precursor state (initiating proliferation). **(B)** If the mRNAs and proteins encoded by Y accumulate faster than those of X in a progenitor, the cell will not differentiate and will have a high proliferation rate. Pathways (microRNA and proteasome pathways) promoting mRNA and protein degradation regulate the process of X and Y accumulation: inefficient mRNA/protein degradation enables rapid X or Y accumulation. **(C)** Vice versa, fast accumulation of X compared to Y enables the progenitor-to-precursor transition. **(D)** However, if the microRNA and 26S proteasome pathways are so effective that they prevent any accumulation of X products, the progenitor would remain in the same state forever (a transition will never occur).

Our hypothesis (based on Dr. Kageyama’s research) that Notch/Hes1-dependent oscillatory gene expression in RPCs prevents them from differentiating requires a mechanism to explain how this oscillatory expression specifically prevents the RPC transition from the proliferative progenitor to the post-mitotic precursor state. Here, we will provide this mechanism, explaining not only how oscillatory expression prevents the transition, but also how this transition happens. We previously described the oscillatory Hes1 GRN in which Hes1 acts as an engine, propelling oscillatory expression across numerous interacting genes in the GRN. We suggest that Hes1 is the only real oscillator in the GRN, its oscillatory expression resulting from the previously described mechanism and includes required negative feedback loops (Figure 2). Meanwhile, oscillatory expression of other genes in this oscillatory GRN depends on oscillatory expression of Hes1: the mRNA expression of direct Hes1 target genes is permitted at moments when the Hes1 protein level is low, and prevented when the Hes1 protein level reaches its maximum (Figure 2). As we mentioned above, since genes directly inhibited by Hes1 also activate or inhibit the expression patterns of their own groups of target genes, this induces oscillatory expression of all genes in the Hes1-dependent GRN. However, the period of this GRN’s gene oscillations is determined by Hes1’s period. Since Hes1 is a real oscillator, observations and mathematical modeling suggest that Hes1 mRNA and protein expression oscillates between fixed minima and maxima (differential boundary conditions) while remaining harmonic for a long time; hence, this should result in a constant average level of Hes1 expression over time (Hirata et al., 2002; Jensen et al., 2003; Monk, 2003; Pigolotti et al., 2007; Tiana and Jensen, 2013). While the expression of GRN genes piloted by oscillatory Hes1 expression also undergo related oscillations, the mRNA and protein expression of these genes have no fixed restrictions imposed on them the way real oscillators do, hence average levels of products of these genes do not have to be static and may increase over time (Bonev et al., 2012; Goodfellow et al., 2014). If the GRN genes’ mRNA and/or protein products are inefficiently degraded (e.g., due to diminished microRNA inhibition and/or decreased 26S proteasome activity), they may gradually accumulate—much like a slowly ticking molecular “clock.” We noted above that published data indicate the oscillatory Hes1 GRN should include genes that inhibit Notch signaling as well as genes that promote cell proliferation. Suppose that one or more genes from the oscillatory Hes1 GRN (designated as X in Figure 4) are required to activate the expression of a certain gene (Z) that inhibits Notch signaling (Figure 4A). We propose that at least one other Hes1 GRN gene (designated as Y in Figure 4) mediates expression of a gene (P) promoting cell proliferation (Figure 4A). However, if Z (P) gene product levels did not reach the required threshold, Z (P) gene products are immediately degraded. Only when the X (Y) level is high enough to promote supra-threshold Z (P) gene product levels in a short period of time, the Z (P) level will be stable and high enough to inhibit Notch signaling (promote cell proliferation; Figure 4A). We hypothesize that the progenitor-to-precursor transition requires a gradual accumulation of oscillating X mRNA and proteins up to a supra-threshold level. When products of X reach the supra-threshold level and sustain that level over a long period of time, they activate stably high Z expression, promoting low and sustained (non-oscillatory) Notch signaling expression, followed by the activation of additional genes specific to retinal neuronal phenotypes (Figure 3). The reduced Notch signaling activity will also generate a positive feedback loop—due to the absence of Hes1-mediated inhibition of X expression—that facilitates the sharp transition from the progenitor state to the precursor state; hence we refer to this as a molecular “switch.” In the section entitled “*Putative mechanisms regulating the transition of retinal cells from the proliferative progenitor state to the post-mitotic precursor state*,” we provided some examples using real genes and published evidence. It should be noted that the longer a progenitor remains in the undivided (non-proliferative) state, the more likely (higher probability) it is for the progenitor to initiate differentiation, since the required genes may have had enough time to reach the supra-threshold level, inhibiting Notch signaling according to the above-mentioned mechanism. Meanwhile, products of Y may also accumulate to reach a required supra-threshold level to activate P and initiate progenitor proliferation (Figure 4A). If a progenitor divides earlier, before the supra-threshold level of X is reached, products of these genes will be diluted in the daughter cells and the accumulation process of X products restarts. We propose that there is competition between these two processes, starting either progenitor proliferation or differentiation (Figures 4B,C). Both processes also determine how long an undifferentiated cell remains in the progenitor state.

The mechanism proposed above affects not only the progenitor’s decision to differentiate or divide, but also affects the size of the developing retina. The progenitor-precursor transition begins when the expression of X reaches supra-threshold levels (a “switch”) in RPC. If the products of X are efficiently degraded, they will accumulate very slowly, allowing RPCs to remain in a progenitor state for a long time, thereby producing a large number of progenitor progeny that form a larger adult retina once differentiated (Figure 4B). Meanwhile, if mRNA and proteins encoded by these genes are inefficiently degraded by microRNA and proteasome pathways, then the gene products will accumulate quickly, facilitating the progenitor’s transition into the post-mitotic precursor state, leading to differentiation (Figure 4C). In the latter case, RPCs have a shorter time to proliferate, and consequently generate a smaller number of progenitors, which when differentiated form a smaller retina. Meanwhile, products of genes promoting the cell cycle can also accumulate (a “clock”) and reach a supra-threshold level (a “switch”) in the same progenitor to initiate proliferation (Figure 4). The stability of mRNA and proteins coded by these genes can affect the proliferation rate of the RPCs. Thus, we hypothesize that the number of proliferating cells (progenitors) in the retina depends on how fast the molecular “clocks” regulating Notch signaling and the cell cycle “tick” inside a progenitor. The interplay between both of these mechanisms during development should determine the final size of the retina (Figures 4B,C).

However, what would happen if the microRNA and 26S proteasome pathways are efficient enough to prevent any accumulation of Notch signaling-inhibiting genes at all? It seems obvious that, in this case, RPCs would not be able to stop proliferating (Figure 4). This is an extreme scenario; the retina in this situation would contain all progenitors and no neurons. However, if this “no clock ticking” scenario happens in a single RPC due to mutations in genes coding the relevant elements of microRNA and proteasome pathways, that single cell could potentially generate a colony of “enemies” waiting for the right moment to attack. Such a colony may not proliferate uncontrollably at first, due to the Hippo contact inhibition pathway (Gumbiner and Kim, 2014). But the “enemy” cells would retain their hidden potential to proliferate, unleashing their fury (i.e., giving rise to retinoblastoma) when additional mutations revoke this restriction. Thus, if our model of retinal neurogenesis is correct, all genes involved in the “clock-switch” models (at least the genes that regulate the degradation of mRNA and proteins encoded by the GRN genes) could plausibly regulate carcinogenesis (retinoblastoma formation) in the retina and could be suggested for targeted therapy. Since oscillatory gene expression determines the proliferative state of progenitors, while sustained gene expression facilitates the transition into a post-mitotic precursor state, treatments that force the transition from oscillatory to sustained (non-oscillatory) gene expression might also be valid targets for precision anticancer therapies. Thus, we cannot rule out that the general concepts of our proposed mechanism—oscillatory gene expression being the most important—for retinoblastoma formation may also help to explain mechanisms of carcinogenesis in other tissues.

## Putative Mechanisms Regulating the Transition of Retinal Cells From the Proliferative Progenitor State to The Post-Mitotic Precursor State

Our model of retinal neurogenesis posits that the transition of proliferative RPCs to the post-mitotic precursor state requires significantly reduced Notch signaling activity (low and sustained/non-oscillatory Hes1 levels), yet the exact mechanisms by which Notch signaling is turned off to initiate neuronal differentiation are still unclear. Here, we describe several putative mechanisms based on our own data and other published evidence.

### The Dll1/Dll3 Model

**Figure 5:**
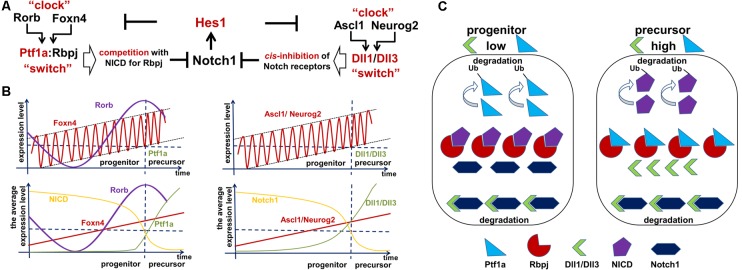
Two Foxn4/Rorb- and Ascl1/Neurog2-based “clock-switch” models may regulate Notch signaling cessation followed by retinal progenitor cell (RPC)-precursor transition. **(A)** The proposed “clock-switch” models that promote increased expression of Ptf1a and Dll1/Dll3 proteins facilitating Notch1 inhibition and RPC differentiation. **(B)** Like a slowly ticking molecular “clock” levels of Foxn4/Rorb and Ascl1/Neurog2 gradually increase. When Foxn4/Rorb and Ascl1/Neurog2 expression levels exceed a critical threshold, they generate the required threshold levels for Ptf1a and Dll1/Dll3 to cease Notch signaling activity (like a molecular “switch” to initiate RPC differentiation). **(C)** Visual representation of the essential proteins in the proposed model. Low levels of Dll1/Dll3 and Ptf1a are found in proliferating progenitors, while high levels of these proteins are seen in cells transitioning into a post-mitotic precursor state.

Notch ligands have been shown to have two activities: *cis*-inhibition of Notch signaling within a cell and *trans*-activation of Notch signaling in neighboring cells (Kageyama et al., 2008b; Sprinzak et al., 2010; del Álamo et al., 2011). We previously reported that isolated RPCs express Notch ligands—Dll1 and Dll3—at significantly higher levels than whole retina populations (Dvoriantchikova et al., 2015). While Dll1 can either *trans*-activate or *cis*-inhibit Notch signaling, Dll3 exclusively promotes *cis*-inhibition (Ladi et al., 2005; Kageyama et al., 2008b; Sprinzak et al., 2010; Chapman et al., 2011; del Álamo et al., 2011). *Cis*-inhibition of Notch signaling by either delta ligand leads to degradation of both the delta ligand and the full-length Notch receptor in lysosomes (“mutual inactivation”; Ladi et al., 2005; Chapman et al., 2011). Sprinzak et al. (2010) demonstrated that the response of Notch1 to the *trans*-ligand is graded, while the response to the *cis*-ligand is very sharp, occurring at a finite ligand threshold independent of *trans*-ligand levels. This unique response to the *cis*-ligand creates an ultrasensitive switch between the progenitor state (high Notch1, low ligand levels) and the precursor state (low Notch1, high ligand levels; Sprinzak et al., 2010). Thus, Dll1 and Dll3 should help mediate the cessation of Notch signaling *via* *cis*-inhibition, allowing RPC transition into a post-mitotic precursor state (Figure 5). However, if the number of Notch receptor proteins inside a progenitor is sufficiently higher than the number of Notch ligands, the ligands will mostly undergo degradation and the cell will remain in a progenitor state (high Notch levels, low Dll1/Dll3 ligand count; Figure 5). But if the number of Notch receptor copies in a progenitor is lower than the number of Notch ligands, the Notch receptors will mostly undergo degradation and the cell will become Notch ligand-positive and transitioning to the post-mitotic precursor state (low Notch levels, high Dll1/Dll3 ligand count; Figure 5). In this model, the transition from the progenitor state to the precursor state in the embryonic retina depends on the levels of Dll1/Dll3 in an RPC. Thus, there is a threshold level—a molecular “switch”—for Notch ligands (Dll1/Dll3) to promote the transition from a proliferating progenitor state to a post-mitotic precursor state *via* a *cis*-inhibition mechanism.

As we noted above, if Dll1/Dll3 levels are sub-threshold, they mostly undergo degradation and cannot accumulate. An additional mechanism is required to push supra-threshold Dll1/Dll3 levels to reduce Notch signaling in RPC and start the progenitor-precursor transition. Notch ligand (Dll1/Dll3) levels are controlled by the transcription factors Ascl1 and Neurog2 in the developing retina (Henke et al., 2009a; Nelson et al., 2009; Hufnagel et al., 2010). Both transcription factors facilitate differentiation of progenitors into neuronal phenotypes (Ohsawa and Kageyama, 2008). Ascl1 also mediates Mfng fringe protein expression, which promotes binding of Notch receptors to delta ligands and can thus facilitate both *trans*-activation and *cis*-inhibition mechanisms (Pollak et al., 2013). Ascl1 and Neurog2 expression levels are also inhibited by the Hes1 transcription factor (Ohsawa and Kageyama, 2008). Since the levels of Dll1 and Dll3 depend on the levels of Ascl1 and Neurog2, the transition from a progenitor state to a precursor state fundamentally depends on Ascl1 and Neurog2 expression in this model (Figure 5). The critical feature of our Dll1/Dll3 model is the gradual increase of Ascl1 and Neurog2 transcription factor levels—like a ticking “molecular clock”—in RPCs, mediating increasingly higher Notch ligand production. Since Hes1 inhibits Ascl1 and Neurog2 expression, and Hes1 expression oscillates in RPCs—according to our model of retinal neurogenesis—then Ascl1 and Neurog2 expression should also oscillate in RPCs (Figure 5B). If Ascl1/Neurog2 mRNA and proteins degrade inefficiently, gradual accumulation of Ascl1/Neurog2 may occur in RPCs. If Ascl1/Neurog2-mediated expression of Notch ligands remains sub-threshold in an RPC, the ligands are mostly degraded and the cell remains in a progenitor state. The sharp transition from the proliferating progenitor state to the post-mitotic precursor state occurs when Ascl1/Neurog2 levels become high enough to mediate supra-threshold expression of Dll1/Dll3, flipping the aforementioned “switch” that initiates the transition to a precursor state (Figure 5). Thus, Ascl1 and Neurog2 have their own threshold level to induce supra-threshold expression of Dll1/Dll3 and promote a transition (Figure 5). We discussed this scenario previously; note that Dll1/Dll3 can be labeled as Z in Figure 4. The rapid degradation of Notch receptors in an RPC creates a positive feedback loop, facilitating even higher Ascl1/Neurog2 production as Hes1-dependent inhibition of Ascl1/Neurog2 expression ceases (Ohsawa and Kageyama, 2008; Shimojo et al., 2008; Imayoshi et al., 2013). Removal of Hes1-mediated inhibition of Ascl1/Neurog2 leads to higher and sustained (non-oscillatory) expression of these genes followed by even higher Dll1/Dll3 expression, eventually making the RPC Notch ligand-positive, mediating the total inhibition of Notch signaling and the transition into a stable, post-mitotic precursor state (Figure 5).

Since Ascl1 and Neurog2 redundantly promote Dll1/Dll3 expression, inactivation of only one gene may have no effect. Meanwhile, a knockout of both genes could result in the total absence of Notch ligands and may disrupt the Notch-mediated lateral inhibition mechanism promoting RPC differentiation. At the same time, the ability to gradually up- or down-regulate Ascl1 and Neurog2 expression using currently existing genetic approaches—like RNA interference technology or Tet-Off/Tet-On inducible systems and animals like tetO-mAscl1-ires-GFP—may support our Dll1/Dll3 model (Jorstad et al., 2017). Conditional knockout of Dll3 directly in retinal progenitors may also be helpful to prove this model.

### The Ptf1a Model

Ptf1a is a transcription factor whose role in Notch signaling regulation has been studied extensively (Beres et al., 2006; Hori et al., 2008; Henke et al., 2009b; Lelièvre et al., 2011). Ptf1a competes with the NICD in binding to Rbpj, the DNA-binding transcriptional effector of the Notch pathway (Beres et al., 2006; Hori et al., 2008; Henke et al., 2009b; Lelièvre et al., 2011). Rbpj interacts with Ptf1a *via* the same domain targeted by Trip12, a Ptf1a-specific E3 ubiquitin ligase that facilitates rapid degradation of Ptf1a (Beres et al., 2006; Henke et al., 2009b; Hanoun et al., 2014). Thus, we hypothesize that binding to Rbpj should prevent Trip12- mediated Ptf1a degradation, while free (unbound) Ptf1a should be quickly destroyed. Since Ptf1a competes with the NICD for Rbpj binding, high Ptf1a levels should facilitate Ptf1a/Rbpj complex formation, freeing the NICD and thereby promoting NICD degradation—*via* targeting of its PEST domain—and preventing Notch signaling activation (Carrieri and Dale, 2016). These events should catalyze the transition from the proliferative progenitor state to the post-mitotic precursor state. Meanwhile, if Notch levels and Notch signaling activity are high, Rbpj will exist predominantly as part of a complex with NICD, leaving unbound Ptf1a to be quickly degraded in the presence of Trip12, according to our hypothesis, and should thus be nearly undetectable in progenitors. The published data support this suggestion (Fujitani et al., 2006). In a manner similar to the Dll1/Dll3 model, we hypothesize that there exists a Ptf1a threshold level—a “switch”—that regulates the transition of RPCs from the progenitor state to the precursor sate (Figure 5). The observation of rosette-like structures—a histologic hallmark of undifferentiated progenitor cell groupings—in the retinas of Ptf1a knockout animals supports the role of Ptf1a as an inhibitor of the progenitor state in the retina (Fujitani et al., 2006).

Much like the Dll1/Dll3 model, Ptf1a is unstable and mostly undergoes degradation at sub-threshold levels. Thus, we need a mechanism to induce supra-threshold Ptf1a expression levels, similar to the manner in which the Dll1/Dll3 model promotes RPC differentiation. Ptf1a levels depend on the activity of Foxn4 and Rorb, a pair of transcription factors that directly activate the Ptf1a promoter in RPCs (Liu et al., 2013). Combining the data above, we suggest a mechanism similar to the Dll1/Dll3 model (Figure 5). We propose that Ptf1a competition with the NICD for Rbpj binding functions like a molecular “switch” to inhibit Notch signaling in RPCs. However, levels of Ptf1a expression must reach or exceed a critical threshold in order for Notch signaling (based on NICD level) to be switched off. The Ptf1a model also posits that, like Ascl1/Neurog2 levels in the Dll1/Dll3 model, Foxn4/Rorb levels gradually accumulate—like another slowly “ticking molecular clock”—driving increasingly higher expression levels of Ptf1a. If the level of Ptf1a in the RPC remains sub-threshold, Ptf1a will be quickly degraded. The switch from the progenitor state to the precursor state in an RPC occurs sharply when Foxn4/Rorb levels become sufficiently high enough to mediate the production of significant—supra-threshold—amounts of Ptf1a. This means that, similar to the Dll1/Dll3 model, Foxn4 and Rorb have their own threshold levels that must be met to induce supra-threshold expression of Ptf1a and promote transition (Figure 5). We discussed this scenario previously with Ptf1a as Z in Figure 4. The high levels of Ptf1a inhibit Notch signaling (due to rapid NICD degradation), facilitating a transition into the post-mitotic precursor state (Figure 5). We hypothesize that Hes1 inhibits (directly or indirectly) Foxn4 expression in RPCs promoting an oscillatory expression of Foxn4 in these cells (Foxn4 belongs to the Hes1-dependent oscillatory GRN). Just like the Dll1/Dll3 model, if Foxn4 mRNA/proteins are degraded inefficiently, gradual accumulation of Foxn4 may occur in the RPC. However, removal of Hes1-dependent inhibition of Foxn4 expression in RPCs may provoke a positive feedback loop, promoting Foxn4 production followed by an increase in Ptf1a expression, mediating the total inhibition of Notch signaling and the transition into a stable, post-mitotic precursor state (Figure 5). It should also be noted that the Ptf1a/Rbpj complex promotes Neurog2 (Ngn2) and Dll1 expression (Henke et al., 2009b; Ahnfelt-Ronne et al., 2012). Thus, the Ptf1a model and the Dll1/Dll3 model may work together in RPCs, promoting a sharp transition from the progenitor state to the precursor state. Similar to what we mentioned in the Dll1/Dll3 model, the ability to regulate Foxn4 and Rorb expression using various genetic approaches should be helpful in proving the model (Jorstad et al., 2017).

### The miR-9 Model

This model is based on a study that used mathematical modeling to evaluate how a GRN comprised of the double-negative interaction between Hes1 and miR-9 influences the transition from an oscillatory progenitor state to a non-oscillatory precursor state (Goodfellow et al., 2014). In this gene network, the Hes1 protein inhibits its own expression as well as the expression of miR-9, a microRNA that inhibits Hes1 production (Bonev et al., 2012; Tan et al., 2012; Goodfellow et al., 2014). Meanwhile, Notch signaling (NICD) activates the expression of both Hes1 and miR-9 (Roese-Koerner et al., 2017). The authors demonstrated that if Hes1-mediated inhibition of miR-9 expression is weak/low, miR-9 is allowed to gradually accumulate, eventually promoting the transition from oscillatory Hes1 expression (the progenitor state) to low and sustained Hes1 expression (the non-oscillatory precursor state; Goodfellow et al., 2014). They also showed that the timing of this transition is a function of the initial amount of miR-9 in a progenitor (i.e., higher initial miR-9 levels facilitate a more rapid transition). Thus, accumulation of miR-9 in RPCs may be as important as the accumulation of Ascl1/Neurog2 (Dll1/Dll3 model) and Foxn4/Rorb (Ptf1a model) in driving the transition from the proliferative progenitor state to the post-mitotic precursor state. Importantly, expression of miR-9 in RPCs over the course of retinal development was shown previously (Karali et al., 2007, 2010; La Torre et al., 2013). Misexpression of miR-9 in RPCs leads to reduced RPC proliferation followed by neuronal differentiation (Hu et al., 2014). However, some level of miR-9 is required to support oscillatory Hes1 expression, while high or low miR-9 levels promote RPC differentiation (Coolen et al., 2013; Goodfellow et al., 2014). Thus, similar to the models above, the ability to regulate miR-9 expression in RPCs is critical to prove this hypothesis. The critical role of miR-9 in neurogenesis was reviewed in Coolen et al. (2013).

### Notch Degradation and the Numb Model

Using mathematical modeling, Barton and Fendrik (2013) analyzed differences in rates of Notch receptor degradation between two neural progenitors coupled by the Delta/Notch pathway (i.e., Notch delta ligand on the surface of one cell *trans*-activating a Notch receptor in a neighboring cell). They found that a slight difference in Notch receptor degradation between two progenitors preserves oscillatory expression of Neurog2, Hes1, and Dll1 in one cell, but halts oscillatory expression of these genes in the other followed by increased Neurog2 and Dll1 expression and reduced Hes1 expression. Meanwhile, if the neighboring cells lose contact with each other, the model showed that both cells halt oscillatory expression of the increasing Neurog2 and Dll1 expression and the decreasing Hes1 expression. These results are a good addition and generalization to the “clock-switch” models described above: for example, asymmetric inheritance of Ascl1/Neurog2 and Foxn4/Rorb could quite plausibly lead to different rates of Notch degradation between two daughter cells, enabling differentiation of one but not the other. The difference between our models and Barton and Fendrik’s model is their suggestion that asymmetric inheritance of Numb (a Notch signaling inhibitor) leads to asymmetric Notch degradation between the two daughter cells. We call this the Numb model. The role of the Numb model in retinal development was already thoroughly investigated (Cayouette et al., 2001; Dooley et al., 2003; Kechad et al., 2012).

## The “*Quantum Mechanics*” of Retinal Phenotypes

In quantum theory, quantum particles are recognized as existing in a superposition of states at the same time. All these states are characterized by different probabilities of occurring until direct observation restricts the probable outcomes to only one state. Erwin Schrödinger, one of the fathers of quantum mechanics, proposed a thought experiment to explain the nature of quantum particles; this is referred to as the *Schrödinger’s Cat* paradox, in which we interpret that a cat in a box with a radioisotope, whose decay triggers the release of a deadly poison, is in a superposition of “alive” and “dead” until an observer opens the box to check. We will apply the concept of “superposition of states” and “probability of occurring” to RPCs and the selection of retinal neuronal phenotypes during RPC differentiation. These concepts are not unique to retinal neurogenesis thanks to groundbreaking research of Dr. Harris’ lab and the study by Gomes et al. (2011). Gomes et al. (2011) demonstrated that RPC division and post-mitotic fate choices of the various neuronal subtypes are “*strikingly consistent with a simple stochastic pattern of behavior in which the decision to multiply or differentiate is set by fixed probabilities*.” The authors also demonstrated that retinal cell fate depends primarily on stochastic choices among available fates at the current stage of retinal development. He et al. (2012; Dr. Harris’ lab) demonstrated that RPCs are equipotent regarding their proliferative potential but subject to stochastic influences to make the decision of generating either two progenitors (PP), a progenitor and a precursor (PD), or two precursors (DD; Chen et al., 2012; He et al., 2012). The results of this study indicate that three modes of RPC division (PP, PD, and DD) are possible at the same time with fixed probabilities. Dr. Harris’ lab also demonstrated that RPCs are equipotent in the ability to differentiate into different retinal cell types, but are subject to stochastic expression of key transcription factors required for each phenotype to make a decision (Chen et al., 2012; He et al., 2012; Boije et al., 2014, 2015). As a result, concepts from probability theory should be utilized to describe the decision of an RPC to differentiate into different retinal cell types (Chen et al., 2012; He et al., 2012; Boije et al., 2014, 2015). The authors of these studies operate with stochastic factors or stochastic expression of key fate-influencing transcription factors, which generate the probabilities of an RPC to proliferate or differentiate into different retinal cell types. The oscillatory gene expression in retinal progenitors can perfectly explain the nature of “stochasticity” and allows us to incorporate these data in our model of retinal neurogenesis.

**Figure 6:**
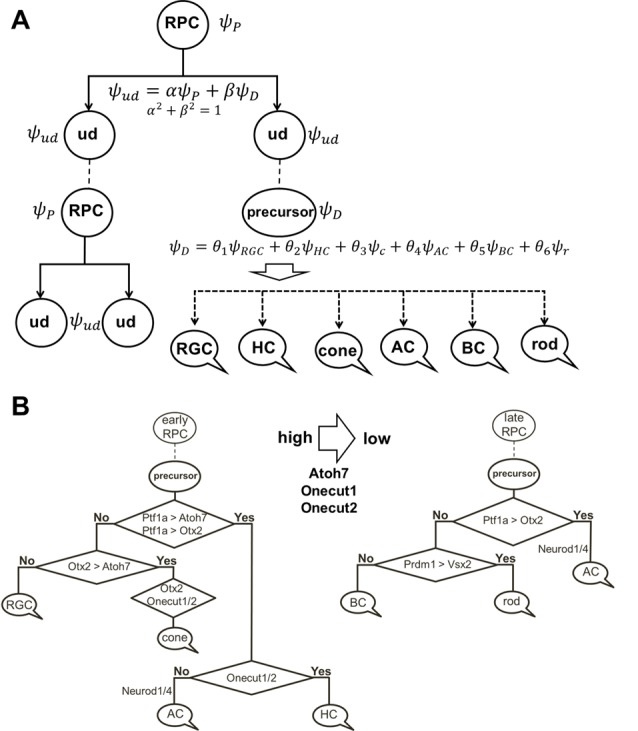
The role of stochastic decisions may be critical in the developing retina. **(A)** Notch/Hes1-powered oscillatory expression of Notch signaling inhibitors, activators/inhibitors of retinal neuronal phenotypes, and cell cycle-promoting genes allows us to consider an undifferentiated (ud) cell in the developing retina as a cell existing in a superposition of progenitor (P) and precursor (D) states at the same time. The undifferentiated cell (ud) can divide (proliferate) with a probability *α*^2^ (and as a result, be recognized as an RPC) or become a precursor (D) with a probability *β*^2^. Meanwhile, oscillatory expression of key genes regulating all retinal neuronal phenotypes and the inheritance of products of these genes from the mother cell after division allows us to consider the post-mitotic precursor (D) in the developing retina as a cell existing in a superposition of states related to all retinal neuronal phenotypes at the same time. **(B)** The decision of a post-mitotic precursor (D) to differentiate into only one of many retinal neuronal phenotypes depends on the level of transcription factors required for this phenotype (which is result of oscillatory gene expression and inheritance from the mother RPC and is, thus, stochastic) during undifferentiated cell (ud)-to-precursor transitions (Notch/Hes1 signaling sustained and low). The probability of a post-mitotic precursor (D) to differentiate into a particular neuronal phenotype depends on the average level of expression of the transcription factors that determine this phenotype during retinal development.

To explain these results, we would like to note that, according to our model of retinal neurogenesis, the oscillatory Hes1-regulated GRN contains Notch signaling inhibitors, activators/inhibitors of retinal neuronal phenotypes, and cell cycle-promoting genes. The expression of all of these genes is present in any single progenitor at the same time. We also proposed that the expression of these genes (with the exception of Hes1) might oscillate and accumulate over time in the progenitor. If the progenitor is gaining products of genes required to reduce Notch signaling activity and promote a progenitor-to-precursor transition (Dll1/Dll3, Ptf1a, and miR-9 models) slowly, relative to products of cell cycle-promoting genes, then this cell will divide and produce two daughter cells (Figure 4). We can then claim that the mother cell that generated the two daughter cells was, indeed, a progenitor (P; “the box was opened, the cat’s state was observed”). Meanwhile, if products of genes required to reduce Notch signaling activity and promote a progenitor-to-precursor transition accumulate (reaching a supra-threshold level) faster than products of cell cycle-promoting genes, then this cell starts the transition into the precursor state without proliferation, and we can claim this cell to be a post-mitotic precursor (D; Figures 4, 6A). Both of these processes compete with each other and the final result depends on the amounts of aforementioned factors inherited from the mother, grandmother, and all other antecedent cells (proliferative ancestry) and the amounts generated in the progenitor itself. Since these events are stochastic in nature, the progenitor’s decision to proliferate or differentiate (without proliferation) can only be described using concepts from probability theory. Thus, a progenitor in the developing retina exists in the “real progenitor” (P) and “precursor” (D) states at the same time until we either observe the cell divide, producing two daughter cells—the mother was a “real progenitor” (P), an RPC—or observe the cell begin the (non-proliferative) transition to the post-mitotic precursor state (D). To avoid confusion with the terms “progenitor” (an undifferentiated retinal cell in states P and D at the same time) and “real progenitor” (state P only), we will substitute “progenitor” (cell in states P and D) with “undifferentiated cell” so that any mention of “progenitor” refers to a “real progenitor” (P state). Thus, we propose that each undifferentiated cell in the developing retina exists in the progenitor (P) state and precursor (D) state at the same time. Each of these states (P and D) has a probability of occurring (Figure 6A). The results of Dr. Harris’ lab and the Gomes’ study support this theoretical observation. We consider two options: (1) the undifferentiated cell divides into two daughter cells, thus observed to have been a progenitor (P); and (2) the undifferentiated cell begins non-proliferative differentiation, and is, thus, a precursor (D).

(1) Suppose that mother cell divides [now we know that it was in the progenitor (P) state] generating two undifferentiated cells. Although newly born undifferentiated daughter cells look alike, at the molecular level they may inherit different amounts (as we noted above) of Ascl1/Neurog2 and Dll1/Dll3 (Dll1/Dll3 model), Foxn4/Rorb and Ptf1a (Ptf1a model), miR-9 (miR-9 model), and Numb (Numb model), as well as cell cycle-promoting factors from the mother progenitor (RPC). Following what is noted above, the decision of one of these daughter cells to proliferate or become a precursor depends on the amounts of aforementioned factors inherited from the mother cell and the amounts generated in the cell over time. Again, since these events are stochastic in nature, the mother RPC exerts no direct control over its daughters’ decision to remain progenitors (P) or to differentiate (D; Figure 6A). Likewise, the two daughter cells do not control each other’s decisions, either (Figure 6A). Thus, the undifferentiated daughter cell fate (P or D) can only be predicted within a set of probabilities that can be obtained experimentally according to Dr. Harris’ lab results and the Gomes’ study (Figure 6A).

(2) As we proposed earlier, Notch signaling-mediated oscillatory expression of Hes1 and its GRN allows the expression of inhibitors (Hes1, Hes5, etc.) and activators [Dll1, Atoh7, Otx2, Ascl1, Neurog2, Foxn4, Ptf1a, Vsx2 (Chx10), etc.] of retinal neuronal phenotypes in an RPC. If the undifferentiated cell decided to differentiate (D state), its fate depends on the respective levels of fate determination factors [Dll1, Atoh7, Otx2, Ascl1, Neurog2, Foxn4, Ptf1a, Vsx2 (Chx10), etc.] stochastically inherited from the mother RPC and generated in a daughter over time (Figure 6A). The fate of the differentiating cell also depends on the stage of retinal development. As we noted in the section titled “*At least two transcription factors are necessary to promote retinal neuronal phenotypes*,” expression of Atoh7 and Onecut1/Onecut2 is very high at the early stage of retinal development and is much lower at the late stage. Vsx2 (Chx10) is a marker of RPCs at the early stage of retinal development, while Vsx2, in combination with Otx2, promotes the BC phenotype at the late stage. Thus, at the early stage of retinal development, if Atoh7 levels exceed levels of Otx2, the cell will initiate the RGC fate program (Figures 3, 6B). If Otx2 levels exceed Atoh7 levels, and if Onecut1/Onecut2 are present, the cell will instead adopt the cone photoreceptor fate (Figures 3, 6B). Sufficiently high levels of Onecut1/Onecut2 act in combination with Ptf1a to initiate the HC fate program (Figures 3, 6B). But if Onecut1/Onecut2 levels are low, Ptf1a acts in combination with Neurod1/Neurod4 to enact the AC fate program. Since expression of Atoh7 and Onecut1/Onecut2 is too low at the late stage of retinal development, the probability for RPC differentiation into RGCs, cone photoreceptors, or HCs is also very low (Figure 6). Meanwhile, if Otx2 expression is still high and combined with high Prdm1 (Blimp1) expression, the rod photoreceptor phenotype is promoted (Figures 3, 6B). At the same time, high Otx2 and Vsx2 (Chx10) expression and low Prdm1 (Blimp1) will initiate the BC phenotype (Figures 3, 6B). Once again, we underscore the fact that all of these fate decisions are stochastically determined and predictable only within a set of probabilities that depend on fate determination factors stochastically inherited from the mother RPC, factors generated in an undifferentiated cell over time, and the stage of retinal development.

As we noted before, the stochastic model of RPC decisions described here is reminiscent of the stochastic nature of a particle’s position, momentum, energy, and higher order observables in quantum mechanics—probabilities only; no absolute, preordained numerical quantities (as Newtonian physics employed) can be used to make predictions. In this regard, much like Schrödinger’s famous cat, an undifferentiated cell in the developing retina may have all possible fates (RPC, RGC, cone or rod photoreceptors, horizontal, amacrine, or BC) with different probabilities (depending on the stage of retinal development) until it actually adopts one (the “box is opened, the cat is observed”). Thus, our oscillatory expression-based model of retinal neurogenesis can perfectly explain the results of Dr. Harris lab and Gomes study (Gomes et al., 2011; Chen et al., 2012; He et al., 2012; Boije et al., 2014, 2015). The possible role of Notch signaling-mediated oscillatory gene expression in stochastic RPC decisions was also suggested in Dr. Harris’ review (Boije et al., 2014).

## Development of MÜller Glia

While our model of retinal neurogenesis posits that low and sustained (non-oscillatory) Hes1 expression is required to promote the transition of RPC into various retinal neuronal phenotypes, in this section, we will argue that high and sustained (non-oscillatory) Hes1 expression is required to promote the MG phenotype in RPC. In differentiating MG, Hes1 is expressed in a high and sustained manner that should prevent the expression of key factors promoting any neuronal phenotype (Furukawa et al., 2000; Ohsawa and Kageyama, 2008; Nelson et al., 2011; Mizeracka et al., 2013). But what mechanism prevents oscillatory Hes1 expression facilitating not reduced, but increased Hes1 mRNA and protein expression in progenitors at the late stage of retinal development? The first mechanism that comes to mind is a classical Notch-dependent lateral inhibition mechanism. If an RPC is surrounded by differentiating neuronal precursors with stable (non-oscillatory) expression of Notch delta (Dll1) ligands permanently exposed on their surfaces, then Notch signaling in this RPC should be constitutively active. If constitutively active, Notch signaling should induce high and sustained (non-oscillatory) Hes1 expression in an RPC; this cell can then start to differentiate into an MG. Importantly, instances of high-density Notch ligand-expressing retinal neuronal precursors in some areas of the retina are quite rare at the early stages of retinal development, but they become very frequent at the end of retinal neurogenesis when many RPCs begin to differentiate into retinal neurons. As a result, this process should promote MG differentiation mostly at the late stage of retinal development. Indeed, the experimental data indicate that peak MG differentiation happens mostly at the late stage of retinal neurogenesis, and constitutively active Notch signaling in late RPCs mediates MG differentiation (Furukawa et al., 2000; Ohsawa and Kageyama, 2008; Nelson et al., 2011; Mizeracka et al., 2013). However, theoretical analysis indicates that constitutively active Notch signaling is necessary, but not sufficient to mediate high and sustained expression of Hes1 in differentiating MG (Hirata et al., 2002; Tiana and Jensen, 2013; Boareto et al., 2017). In particular, constitutively active Notch signaling cannot eliminate oscillatory Hes1 expression if the transcriptional and translational time delays are greater than the half-lives of the Hes1 mRNA and proteins (Jensen et al., 2003; Monk, 2003; Swinburne et al., 2008; Takashima et al., 2011). But if the Hes1 mRNA and proteins’ half-lives are increased to several hours, Hes1 can accumulate and achieve high, stable levels (Jensen et al., 2003; Monk, 2003; Swinburne et al., 2008; Takashima et al., 2011; Goodfellow et al., 2014). Goodfellow et al. (2014) demonstrated that the cross-repressive interaction between Hes1 and miR-9 (a microRNA) leads to bistability, resulting in either stably low (miR-9-model) or stably high Hes1 levels. The authors demonstrated that a non-oscillatory (stable) state with high Hes1 levels is the result of strong repression of miR-9 expression by the Hes1 protein. Thus, an additional mechanism is required to promote the Notch-mediated MG phenotype in the developing retina.

**Figure 7:**
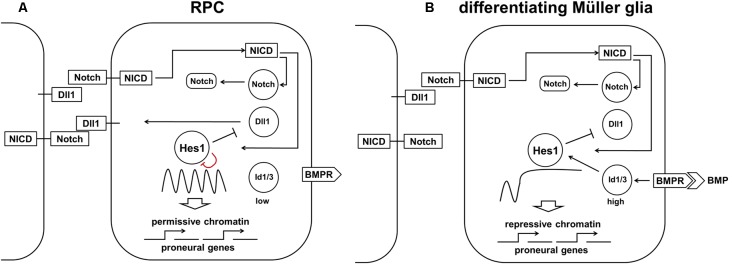
The proposed model requires both high Notch signaling activity and high Id protein family production to promote the high and sustained Hes1 expression that facilitates Müller glia (MG) differentiation. **(A)** Constitutively active Notch signaling cannot, alone, eliminate oscillatory Hes1 expression, so an additional mechanism is required to promote the RPC transition into MG. **(B)** A transient wave of BMP expression in the developing retina promotes high Id protein family expression which, in combination with constitutively active Notch signaling, mediates high and sustained Hes1 expression, followed by complete repression of pro-neuronal factors in differentiating MG. The ability of Hes1 to promote the transition of chromatin from a permissive to a repressive state in promoters of target genes may prevent the expression of pro-neuronal genes stabilizing the MG phenotype.

Two studies (experimental and theoretical) demonstrated that interaction between Notch/Hes1 signaling and the Id protein family promote high and sustained Hes1 expression followed by complete repression of pro-neuronal expression (Bai et al., 2007; Boareto et al., 2017). The members of the Id (Inhibitor of differentiation/DNA binding) family are helix-loop-helix (HLH) proteins that lack a basic (b) DNA-binding domain (Perk et al., 2005; Bai et al., 2007). These proteins, which form heterodimers with pro-neuronal bHLH transcription factors and prevent them from binding to DNA, act as dominant-negative regulators of neuronal fate (Yokota, 2001; Bai et al., 2007; Boareto et al., 2017). Since Hes1 proteins are bHLH transcription factors themselves, Id proteins can form heterodimers with Hes1 and inhibit its ability to bind to DNA (Bai et al., 2007; Boareto et al., 2017). However, while most bHLH transcription factors bind to the canonical enhancer box (E-box), Hes1 binds to the N-box and C-site to inhibit gene transcription (Sasai et al., 1992; Takebayashi et al., 1994; Van Doren et al., 1994; Chen et al., 1997; Hirata et al., 2002; Jones, 2004; Bai et al., 2007; Boareto et al., 2017). The Hes1 promoter contains three N-box regulatory elements. Meanwhile, the Hes1 protein inhibits transcription of pro-neuronal genes using C-sites. Bai et al.’s (2007) results suggest that Hes1 binds with higher affinity to the C-site than to the N-box. As a result, Id proteins must reach a higher level in the cell to prevent Hes1 binding to the C-site compared to its N-box (Bai et al., 2007). Thus, at some level, Id proteins can release negative autoregulation of the Hes1 promoter, but cannot prevent Hes1’s inhibition of pro-neuronal factors with promoters that contain Hes1-regulated C-sites. The absence of Hes1 feedback repression on its own promoter allows high and sustained (non-oscillatory) Hes1 expression (Bai et al., 2007; Boareto et al., 2017) It was shown that misexpression of Id in progenitors inhibits neuron-specific gene expression but upregulates Hes1 expression (Cai et al., 2000; Bai et al., 2007). It should be noted that Id proteins are very unstable (easily degradable), and hence their effects can quickly fade as a result of reduced Id expression (Perk et al., 2005; Bai et al., 2007). Thus, we hypothesize that high Notch signaling activity and increased expression of the Id family in late RPCs promote the transition of these cells from the progenitor to glial (MG) state through two mechanisms: (1) by driving high and sustained (non-oscillatory) expression of Hes1 in RPCs; and (2) by blocking (directly and indirectly due to Hes1 activity) the function of pro-neuronal bHLH transcription factors (Figure 7). We hypothesize that high Id-promoted Hes1 levels should significantly reduce the proliferative activity of differentiating MG since high Hes1 levels inhibit proliferation (Castella et al., 2000; Baek et al., 2006; Kageyama et al., 2008a; Shimojo et al., 2008; Noda et al., 2011). Hes1 can also promote transitioning of chromatin in target genes (genes of neuronal phenotypes and genes promoting cell proliferation) from a permissive to a repressive state, preventing their expression later on, stabilizing the MG phenotype (Figure 7; Davis and Turner, 2001; Takata and Ishikawa, 2003; Kageyama et al., 2007a; Li and Arnosti, 2011; Kok and Arnosti, 2015).

There is plenty of evidence indicating the important role of the Id family in MG development. Members of the Id protein family are expressed in RPCs but are even more highly expressed in differentiating MG (Nelson et al., 2011; Mizeracka et al., 2013; Dvoriantchikova et al., 2015; Ueki et al., 2015). It is a well-established fact that Id protein family expression is mediated by BMP signaling (Miyazono and Miyazawa, 2002; Ueki et al., 2015). Dr. Reh laboratory demonstrated that transient activation of BMP signaling at the end of retinal neurogenesis promotes Id family expression to drive complete repression of neuronal gene expression in differentiating MG, thereby promoting the glial phenotype (Ueki et al., 2015). Dr. Cepko lab demonstrated that misexpression of Id1 and Id3 in the developing retina promotes MG production, while reduced Id1/Id3 expression was associated with reduced MG numbers (Mizeracka et al., 2013). However, the role of Hes1 (and Hes5) in promoting and stabilizing the MG phenotype requires further research using various types of approaches, including epigenetic (ChIP-seq analysis, etc.).

## Discussion

Combining all the published data and hypotheses above, we can now propose an integrated model of neurogenesis in the developing retina. We propose that at the early stage of embryogenesis, a group of undifferentiated cells in the optic vesicle—in which the Notch/Dll1-mediated lateral inhibition mechanism prevails over the Notch/Jag1-mediated lateral induction mechanism—are the founders of the future retina. While lateral induction promotes only one phenotype, Notch/Dll1-mediated lateral inhibition allows the generation of a huge variety of cell types in the developing retina by jumpstarting oscillatory Hes1 expression in undifferentiated cells. In turn, Hes1 activates oscillatory expression in its GRN containing Notch signaling inhibitors, activators/inhibitors of retinal neuronal phenotypes, and cell cycle-promoting genes. Oscillatory Hes1 expression allows the expression of all these genes in the same undifferentiated cell at the same time. However, the expression levels of these genes fluctuate over time, modulated by Hes1 oscillations within the cell. The levels of Notch signaling inhibitors, activators/inhibitors of neuronal phenotypes, and cell cycle-activating genes not only oscillate, but also accumulate in the undifferentiated cell, and depending on which factors accumulate fast enough to reach their required supra-threshold levels (Notch signaling inhibitors or cell cycle activators), the undifferentiated cell starts to differentiate without further proliferation, becoming a precursor (D), or it divides, concluding that it was a progenitor (P). If an undifferentiated cell divides, the factors above may become diluted in daughter cells and the process of accumulation restarts. Because of the stochastic nature of these events, probabilities are necessary to describe the process of selecting a single outcome (P or D; results of Dr. Harris lab, Gomes and colleagues). Thus, before a decision is made, the undifferentiated cell may be interpreted as existing in both states (P and D) simultaneously with each state assigned a probability of occurring. If the undifferentiated cell starts to differentiate (D), the selection of one neuronal phenotype out of many other possibilities depends on the level of transcription factors required for the selected phenotype. Because these factors may be inherited at different levels from the mother cell after proliferation, and because these factors may oscillate and accumulate at different levels in the post-mitotic precursor (D), the process of selection when inhibitory Notch signaling activity is ceased is purely stochastic and requires probabilistic approaches to describe the selection process (results of Dr. Harris lab, Gomes and colleagues). In a manner analogous to quantum mechanics, probabilities can be used to predict these cell transitions, which depend on average expression levels of phenotype-determining transcription factors in undifferentiated cells (RPCs) during retinal development. However, these probabilities may experience time-dependent fluctuations during retinal development—reduced expression of Atoh7, Onecut1, and Onecut2 in the developing retina reduces the probability of retinal progenitor differentiation into early-born neurons (RGCs, cone photoreceptors, and HCs) during the late stage of retinal neurogenesis.

While low and sustained (non-oscillatory) Notch signaling activity is required to promote the transition of retinal progenitors into various retinal neuronal phenotypes, high and sustained (non-oscillatory) Notch signaling activity synergized with high BMP signaling-induced Id protein family production is required to promote the MG phenotype in progenitors during the late stage of retinal development. This model is based on results obtained by Dr. Cepko’s and Dr. Reh’s labs. Meanwhile, if oscillatory gene expression in some undifferentiated cells/RPCs does not stop due to pathological events (e.g., mutations in genes regulating the stability of mRNA and proteins of genes like Notch1, Hes1, Dll1, Ascl1, Neurog2, etc.), these cells may begin retinoblastoma formation.

## Conclusions

In this article, we have outlined a model that accounts for retinal neurogenesis from a fresh perspective. Unlike previous models that have required the existence of many intrinsically different RPCs, the model proposed here is based on Dr. Kageyama’s oscillatory gene expression paradigm and Dr. Harris’ concept on the contribution of stochasticity to the proliferation and fate choice of retinal progenitors, allowing us to explain retinal neurogenesis using only early and late RPC types. Regarding our model, observed RPC heterogeneity arises from oscillatory gene expression within these two types of RPCs. While oscillatory gene expression prevents the differentiation of progenitors into retinal phenotypes, it creates the foundation for the observed stochasticity in RPCs’ decision to either proliferate or differentiate (in this case, the fate choice is also stochastic). Thus, if our model is correct, certain core concepts in the field of ophthalmology would be fundamentally modified: in particular, any vestige of the old paradigm that depicts gene expression as a static process will be discarded, supplanted by more accurate, data-rich models built on the observation that gene expression is inherently dynamic in nature. This dynamic gene expression milieu arises from complex GRNs that contain myriad feedback loops (negative and positive). Importantly, future studies will necessitate the combination of theoretical and experimental approaches to achieve success, since oscillatory gene expression is often too complicated to analyze *in vivo*, the experimental process should be predicated on building mathematical models and evaluating the models’ predictions experimentally.

## Data Availability Statement

The datasets generated for this study are available on request to the corresponding author.

## Author Contributions

DI wrote the manuscript.

## Conflict of Interest Statement

The author declares that the research was conducted in the absence of any commercial or financial relationships that could be construed as a potential conflict of interest.
